# Diversity and Ecological Potentials of Marine Viruses Inhabiting Continental Shelf Seas

**DOI:** 10.1002/advs.202511707

**Published:** 2025-11-14

**Authors:** Xiaoyue Guo, Yantao Liang, Chen Gao, Hao Yu, Meiwen Wang, Hongbing Shao, Yeqing Yang, David Paez‐Espino, Andrew McMinn, Min Wang

**Affiliations:** ^1^ MoE Laboratory of Evolution and Marine Biodiversity College of Marine Life Sciences Institute of Evolution and Marine Biodiversity Frontiers Science Center for Deep Ocean Multispheres and Earth System Center for Ocean Carbon Neutrality Ocean University of China Qingdao 266003 China; ^2^ Haide College Ocean University of China Qingdao 266100 China; ^3^ College of Marine Resources and Environment Hebei Normal University of Science and Technology Qinhuangdao 066004 China; ^4^ College of Marine Science and Technology China University of Geosciences (Wuhan) Wuhan Hubei 430074 China; ^5^ UMT‐OUC Joint Centre for Marine Studies Qingdao 266003 China; ^6^ Ancilia Biosciences Inc. New York NY 10032 USA; ^7^ Institute for Marine and Antarctic Studies University of Tasmania Hobart TAS 7001 Australia

**Keywords:** continental shelf seas, diversity, ecological potentials, spatiotemporal dynamics, viromes

## Abstract

Viruses are critical components of all marine ecosystems. However, the role of viruses in continental shelf seas, which are usually areas of intense economic development, remain poorly characterized. Here, 62 seawater viromes from the eastern continental shelf seas of China (ECSSC), collected between 2017 and 2022, are systematically analyzed. A total of 310,628 viral operational taxonomic units (vOTUs) are identified, providing the most comprehensive overview of DNA viral communities in the ECSSC. Among them, 136,652 (42.3%) vOTUs could be classified into 89 families, dominated by *Kyanoviridae*, *Autographiviridae* and *Zobellviridae*. Only 31,994 (10.3%) of the vOTUs have predicted putative hosts, of which 91.4% are associated with specific hosts, including 31 bacterial and 10 archaeal phyla. The uniqueness and novelty of viruses from the ECSSC are identified and found to be much greater than those from GOV 2.0 and other environmental viromes. Viral communities undergo significant seasonal changes in different seasons and regions of the ECSSC. Here, 20,146 auxiliary metabolic genes are identified, of which carbohydrate, sulfur, and photosynthetic pathways are significantly enriched in the ECSSC compared to other marine ecosystems. This study establishes a foundational baseline for seawater viruses in the ECSSC, highlighting viral diversity and its potential impact on the biogeochemistry of continental shelf sea ecosystems.

## Graphical abstract

To the best of the knowledge, this study provides the largest viral genome dataset from a continental shelf sea. It reveals that over half of vOTUs are novel, highlighting the remarkable viral novelty in the eastern continental shelf seas of China (ECSSC). Viral communities exhibit pronounced seasonal and regional shifts, reflecting environmental heterogeneity. A total of 20 146 AMGs are identified, with those involved in carbohydrate, sulfur, and photosynthetic pathways being significantly enriched in the ECSSC.

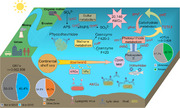

## 1 Introduction

Continental shelf seas represent some of the most productive marine ecosystems and play crucial roles in global climate change. They serve as essential ecological ecotones between terrestrial environments and the open ocean.^[^ ^1^ ^]^ These highly productive regions face intense anthropogenic threats—including coastal urbanization, industrialization, agriculture, and pollution, which drive excessive nutrient loading, organic matter accumulation and cause chemical and biological contamination. These impacts together threaten their ecological sustainability.^[^ ^2^ ^]^ The current flow of total nitrogen into rivers from human waste and agricultural runoff is likely to double.^[^ ^3^ ^]^ Continental shelf eutrophication, caused by nitrogen and phosphorus enrichment, promotes phytoplankton blooms, including harmful algal species, with cascading impacts on marine ecosystems.

Viruses are the most abundant and genetically diverse biological entities in the ocean,^[^ ^4^, ^5^, ^6^ ^]^ driving microbial evolution primarily through lysogenic conversion and transduction. Viral lysis reshapes microbial communities and facilitates carbon transfer via the viral shunt, releasing ≈10 billion tons of carbon daily—a process critical to ocean productivity.^[^ ^7^ ^]^ Furthermore, viruses can influence host metabolism by expressing auxiliary metabolic genes (AMGs) during infection.^[^ ^8^ ^]^ Marine viral diversity and functional potential have advanced markedly in recent decades, driven by high‐throughput sequencing and bioinformatics.^[^ ^9^ ^]^ The Global Ocean Virome 2.0 (GOV 2.0) dataset—derived from 145 *Tara* Oceans viromes—identified 488130 viral operational taxonomic units (vOTUs; ≥5 kb or circular contigs, including 195728 ≥10 kb), expanding global ocean DNA viral diversity by 12‐fold and establishing the largest marine viral genome resource to date.^[^ ^10^ ^]^ Subsequent analysis of 7.6 Tb of *Tara* Oceans paired prokaryotic and viral metagenomes increased the number of vOTUs to 579904 (16% rise) and identified 86913 auxiliary metabolic genes (AMGs) grouped into 22779 gene clusters, with 7248 (≈32%) representing novel functions.^[^ ^11^ ^]^ Viral AMGs were concentrated in metabolic “hot spots,” dominating nine pathways (e.g., carbohydrate, amino acid, and nucleotide metabolism) and outnumbering host homologs in lipid A phosphate and phosphatidylethanolamine biosynthesis.^[^ ^12^ ^]^ Recently, seasonal changes in the abundance of 7957 Antarctic vOTUs further underscored the important viral roles in Southern Ocean dynamics under climate change.^[^ ^13^ ^]^ Despite these advances, seawater viruses in continental shelf seas remain understudied.

The eastern continental shelf seas of China (ECSSC), encompassing the Bohai Sea, Yellow Sea, and East China Sea, are an important ecosystem under significant anthropogenic pressure. As one of Earth's largest continental shelf systems, the ECSSC receives substantial nutrient and antibiotic inputs from densely populated catchments,^[^ ^14^, ^15^ ^]^ creating conditions that favor both coastal eutrophication and the potential proliferation of antibiotic resistance genes (ARGs).^[^ ^16^, ^17^, ^18^ ^]^ In addition, the transduction of virus‐encoded virulence factors (VFs) is widely considered as a major factor in bacterial pathogenesis. The presence of ARGs and VFs in viral populations is of particular ecological concern due to their enhanced environmental persistence, rapid replication potential, and broad host range.^[^ ^19^, ^20^ ^]^ Therefore, it is important to understand whether viruses can help bacterial pathogens disseminate ARGs and VFs, especially in the ECSSC where human activity is intense.

**Figure 1:**
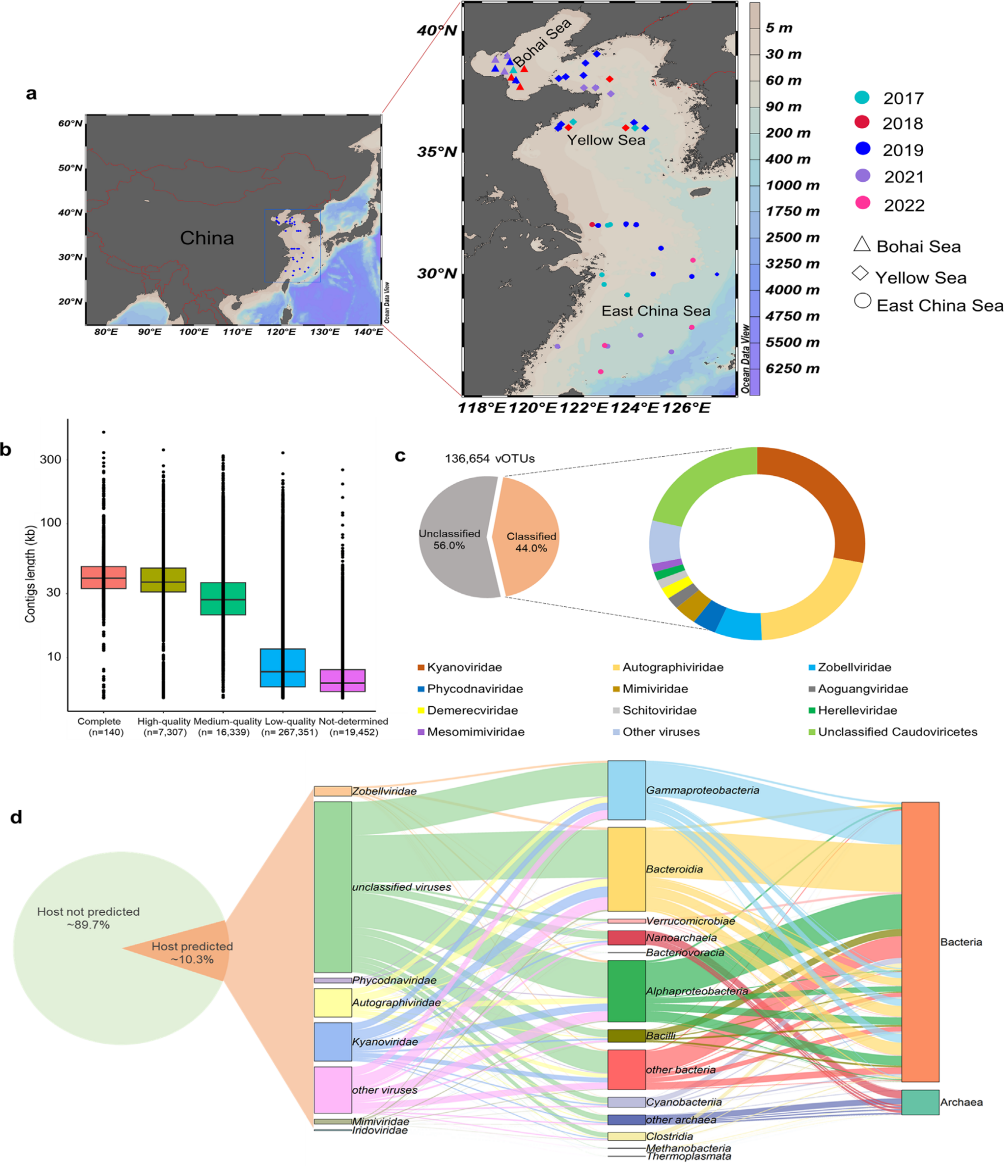
Overview of viruses in the eastern continental shelf seas of China (ECSSC). a) The geographical distribution of the virome sampling sites involved in this study. The sampling location map was created in Ocean Data View v5.7.2 (Schlitzer, Reiner, Ocean Data View, https://odv.awi.de, 2021). b) Distribution of the completeness of viral operational taxonomic unit (vOTUs) in the ECSSC virome dataset. c) Proportion of taxonomic families identified in the ECSSC viral populations. d) Predicted host‐virus interactions. Percentage and taxonomy of vOTUs for which a host was predicted are shown on the left; the taxonomy of predicted hosts is shown on the right.

Here, we present a comprehensive analysis of the diversity and ecological potential of marine viruses in the ECSSC (**Figure** **1a**). A high‐quality viral genome dataset comprising 310628 vOTUs with length ≥5 kb or ≥1.5 kb and circular, including 136653 vOTUs ≥10 kb was generated. This dataset, derived from 62 virome samples collected across the ECSSC from 2017 to 2022, represents the largest marine viral genome dataset from continental shelf seas. The viral community structure and diversity was characterized, virus‐host interactions and the ecological roles of viruses in the ECSSC was explored and the influence of environmental factors on viral communities and functional dynamics was assessed.

## 2 Results

### 2.1 The Eastern Continental Shelf Seas of China Viromes (ECSSCV) Dataset

A large‐scale ECSSCV dataset, derived from 2.92 Tb sequencing of 62 virome samples, was collected between 2017 and 2022 (Figure 1a, Table S1, Supporting Information); this accounts for 73.9% of the Global Ocean Viromes 2.0 (GOV 2.0) dataset.^[^ ^10^ ^]^ A total of 7659737 viral contigs (≥1.5 kb) and 3112364 viral contigs (≥10 kb) were identified using the combination of VirSorter2, DeepVirFinder, and VIBRANT. After clustering (95% average nucleotide identity), 779410 vOTUs (≥1.5 kb) were generated, including 310628 vOTUs (≥5 kb or ≥1.5 kb and circular) and 121698 vOTUs (≥10 kb) (Figure S1a, Supporting Information), accounting for 63.6% of 488130 vOTUs and 62.2% of 195728 vOTUs in the GOV2.0, respectively.^[^ ^10^ ^]^ It was found that the average mapped ratio of 62 virome samples was 75.26%, indicating that the viral contigs we used for analysis could represent the viromes. Notably, 140 complete and 7307 high‐quality viral genomes (Figure 1b) were recovered, representing a 79.9% increase compared to the previously reported coastal dataset (9322 vOTUs) in IMG/VR 4.0.^[^ ^21^ ^]^ The ECSSCV dataset established in this study is apparently the largest DNA viral genome repository from continental shelf seas.

Taxonomic assignment of vOTUs showed that 228289 of 310628 vOTUs (≈73.5%) could be classified to known taxa, of which Duplodnaviria was dominant, followed by Varidnaviria (Figure S1b, Supporting Information). Uroviricota dominated at the phylum level (83.4%), while nucleocytoplasmic large DNA viruses (NCLDVs) comprised 5.64% of the community (Figure S1c, Supporting Information). At the family level, 173974 (≈56.0%) vOTUs remain unclassified, underscoring the substantial unexplored diversity in the ECSSC. Of the classified virus families, 89 were identified, mainly *Kyanoviridae*, *Autographiviridae* and *Zobellviridae* (Figure 1c). Lytic virus lifestyle was found to dominate over lysogenic types (Figure S1d, Supporting Information). The numbers of vOTUs reached saturation at ≈10 samples (Figure S2, Supporting Information), which suggests that ECSSCV adequately represents seawater viromes in the ECSSC.

Only 31994 (10.3%) vOTUs could be linked to putative hosts (Figure 1d), of which 91.4% were associated with specific hosts, consistent with the reported narrow host ranges of marine viruses.^[^ ^22^, ^23^ ^]^ Putative hosts spanned 33 bacterial and 10 archaeal phyla (Figure S3, Supporting Information) with *Pseudomonadota*, *Bacteroidota*, and *Bacillota* emerging as the most widespread bacterial hosts, while Nanoarchaeota and Thermoplasmatota dominated archaeal associations. At the class level, Bacteroidia, Gammaproteobacteria and Alphaproteobacteria were identified as the most frequently putative hosts (Figure 1d). These groups were among the most abundant and active prokaryotic lineages in coastal seas.^[^ ^24^ ^]^ In addition, 56.1% of host‐associated vOTUs remained unclassified at the family level, further indicating a wide range of uncharacterized viral diversity in the ECSSC.

### 2.2 Novelty of Viral Genomes and Genes of ECSSCV in a Global Context

**Figure 2:**
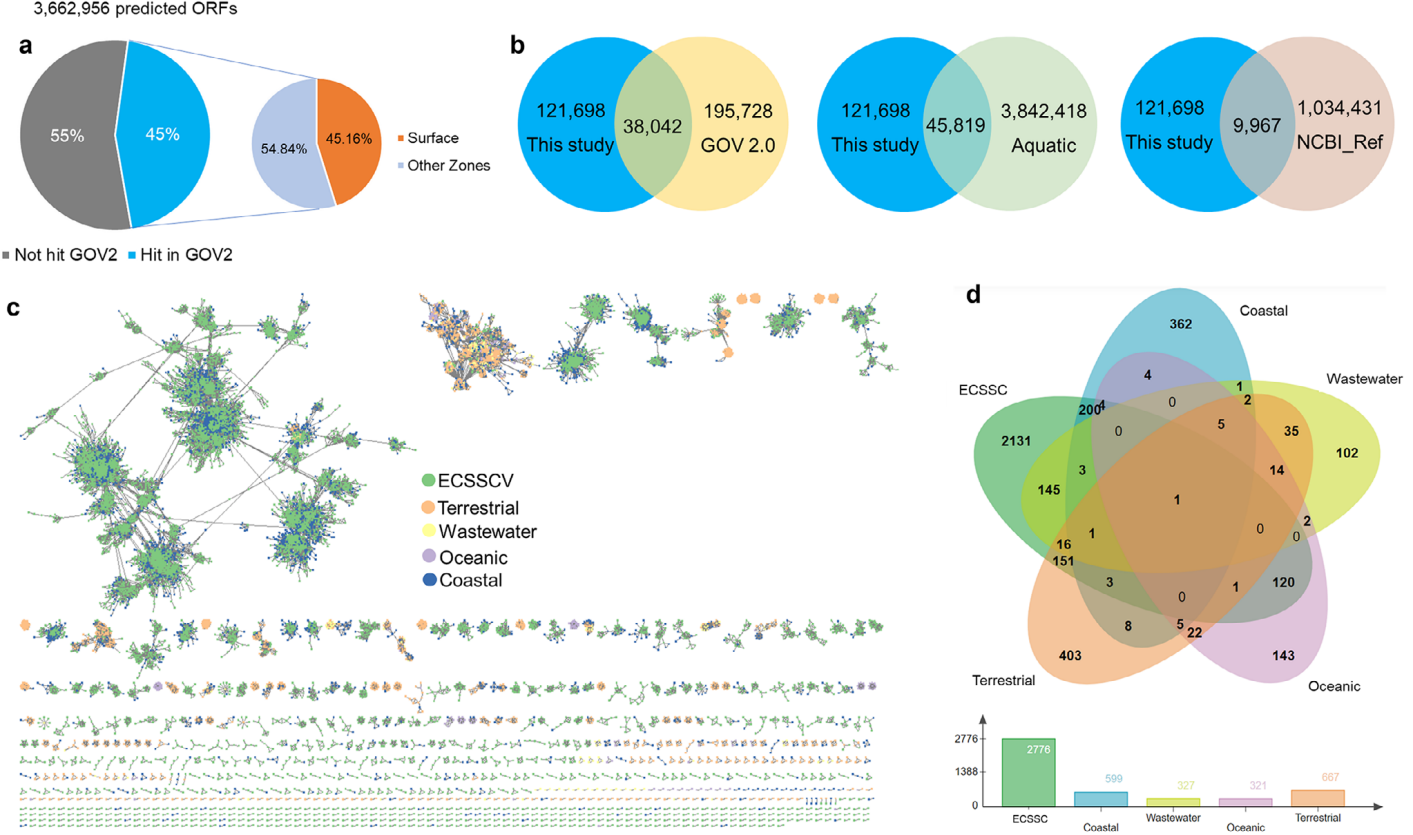
Biogeography of viruses in the ECSSC. Venn diagram showed the number of virus clusters of 62 viromes in the ECSSC of this study and those from other databases, including a) GOV2.0 database, b) IMG/VR v4 aquatic virus database and c) NCBI reference sequence virus database. d) Gene search similarity results shown in pie diagrams were carried out with ORFs (3662956) predicted by vOTUs (≥10 kb) from ECSSC viruses and the rest of viruses from GOV 2.0 Tara viromes data. e) The network of shared predicted proteins content among vOTUs (≥10 kb) of ECSSCV and viral genomes from IMG/VR 4.0 data set covering different habitats, including coastal, terrestrial, wastewater and oceanic. Nodes (circles) represent viral genomes and vOTUs, and the shared edges (lines) indicate shared gene content. Different colors represent different habitats. f) Venn diagram of shared and specific viral clusters (VCs) among the five different environmental habitats.

Cluster analysis of 121698 vOTUs (≥10 kb) from the ECSSC with global viral datasets found limited overlap with established viral sequences. Only 31.26% (38042) of vOTUs clustered with sequences in the GOV 2.0 databases (**Figure** **2**a), while 37.65% (45819) vOTUs shared ≥95% nucleotide identity with IMG/VR v4 aquatic viruses (Figure 2b)—indicating low global similarity. Just 8.19% (9967 sequences) aligned with NCBI RefSeq viral references, underscoring the potential novelty of ECSSC DNA viruses (Figure 2c). Through mapping against GOV 2.0 reads, we found that the proportion of unmapped vOTUs was quite high: 70.24% (85480) in the bathypelagic (BATHY) group, 54.05% (65781) in the Antarctic (ANT) group, and 27.01% (32861) overall (Figure S4, Supporting Information). The novelty may reflect under sampling in other datasets or low‐abundance viruses that fail to assemble.

To place the viral communities in the ECSSC within the global ocean, 3662956 viral genes predicted from vOTUs (≥10 kb) were compared with the GOV 2.0, encompassing Arctic (ARC), Antarctic (ANT), bathypelagic (BATHY), temperate and tropical epipelagic (TT‐EPI), and mesopelagic (TT‐MES) zones. It was found that 55% of the predicted viral genes in the ECSSCV lacked homology to known viral databases, while 45% exhibited homology primarily to open reading frames (ORFs) from viruses in epipelagic zones (Figure 2d). Within the GOV 2.0 database, most matches were to surface viruses (45%), followed by deep‐chlorophyll maximum (29%) and mesopelagic (19%) zones, indicating that ECSSCV viruses were mostly adapted to coastal environments and surface communities.

The ECSSC viral genomes (≥10 kb) were further compared with environmental viromes from the Integrated Microbial Genomes/Viruses (IMG/VR) v4 dataset using a viral protein‐sharing network. Notably, 64.76% of ECSSC viruses were singletons or outliers, showing no connections to known viruses or viral clusters (Figure 2e). Of the 4690 viral clusters (VCs) identified, 2776 were associated with ECSSCV vOTUs (Figure 2f). Most VCs (2131; 76.77%) were endemic to the ECSSCV, while 645 (23.23%) clustered with sequences from other habitats, including coastal (200 VCs), terrestrial (151 VCs), wastewater (145 VCs), and oceanic (120 VCs) environments (Figure 2f). These findings suggest that a significant proportion of ECSSCV viruses are unique, potentially representing novel viral families.

### 2.3 Diversity and Spatiotemporal Variation of Viruses in the ECSSC

**Figure 3:**
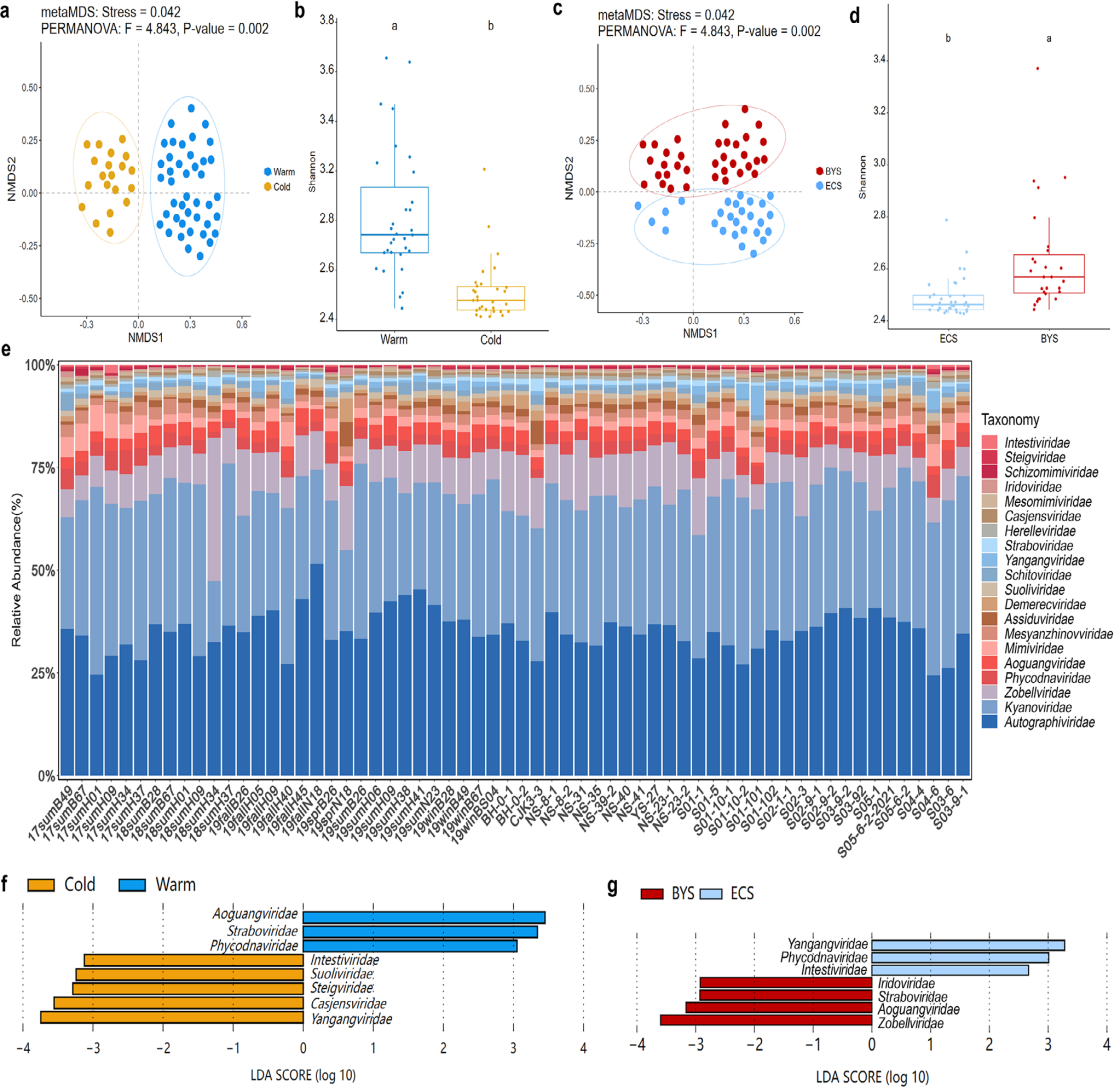
Analyses of the taxonomic composition and diversity of viral community in the ECSSC. a) Taxonomic composition of viral community at family level. b) Shannon index analysis of viral communities in the ECSSC of different seasons. Data are evaluated by Kruskal‐Wallis test. c) Nonmetric multidimensional scaling (NMDS) of viral β‐diversity based on the Bray‐Curtis dissimilarity matrix calculated from the relative abundances of vOTUs during different seasons. PERMANOVA showed the statistical significance of viral community composition. d) LEfSe analysis of viral community at family level during different seasons. e) Shannon index analysis of viral communities in the ECSSC across different regions (BYS: Bohai and Yellow seas; ECS: East China Sea). f) NMDS of viral β‐diversity based on the Bray‐Curtis dissimilarity matrix calculated from the relative abundances of vOTUs across different regions. g) LEfSe analysis of viral community at family level across different regions.

The seasonal variations in viral diversity in the ECSSC were investigated, defining cold (October‐next March) and warm (April‐September) seasons. The results of non‐metric multidimensional scaling (NMDS) found differences in the beta diversity between cold and warm seasons (*P* < 0.01; **Figure** **3**a). The Shannon index in the warm season was significantly higher than in the cold season (Figure 3b). Comparative analysis of viral diversity across the Bohai and Yellow Seas (BYS) and East China Sea (ECS) regions revealed distinct biogeographic patterns. NMDS analysis identified distinct regional clustering of viral communities (*P* < 0.01; Figure 3c). Shannon diversity indices confirmed significant higher viral diversity in the BYS compared to ECS (Figure 3d).

The dominant viral families in the ECSSCV were found to be *Kyanoviridae*, *Autographiviridae* and *Zobellviridae*, followed by *Phycodnaviridae*, *Aoguangviridae*, *Mimiviridae*, and *Mesyanzhinovviridae* (Figure 3e). Linear discriminant analysis effect size (LEfSe) identified pronounced seasonal differences. Specifically, *Aoguangviridae*, *Straboviridae*, *Phycodnavirdae*, and *Zobellviridae* were significantly enriched in warm seasons, while *Kyanoviridae, Demerecviridae, Herelleviridae, and Pachyviridae* were significantly enriched in cold seasons. Regional LEfSe analysis further found that *Zobellviridae*, *Aoguangviridae*, and *Iridoviridae* were enriched in BYS viral communities, whereas *Yangangviridae*, *Bacilladnaviridae, Phycodnaviridae*, and *Pachyviridae* were present in the ECS viral communities (Figure 3g). These results demonstrate significant seasonal and regional restructuring of viral communities in the ECSSC.

### 2.4 Viral AMGs Drive Biogeochemical Dynamics in the ECSSC

**Figure 4:**
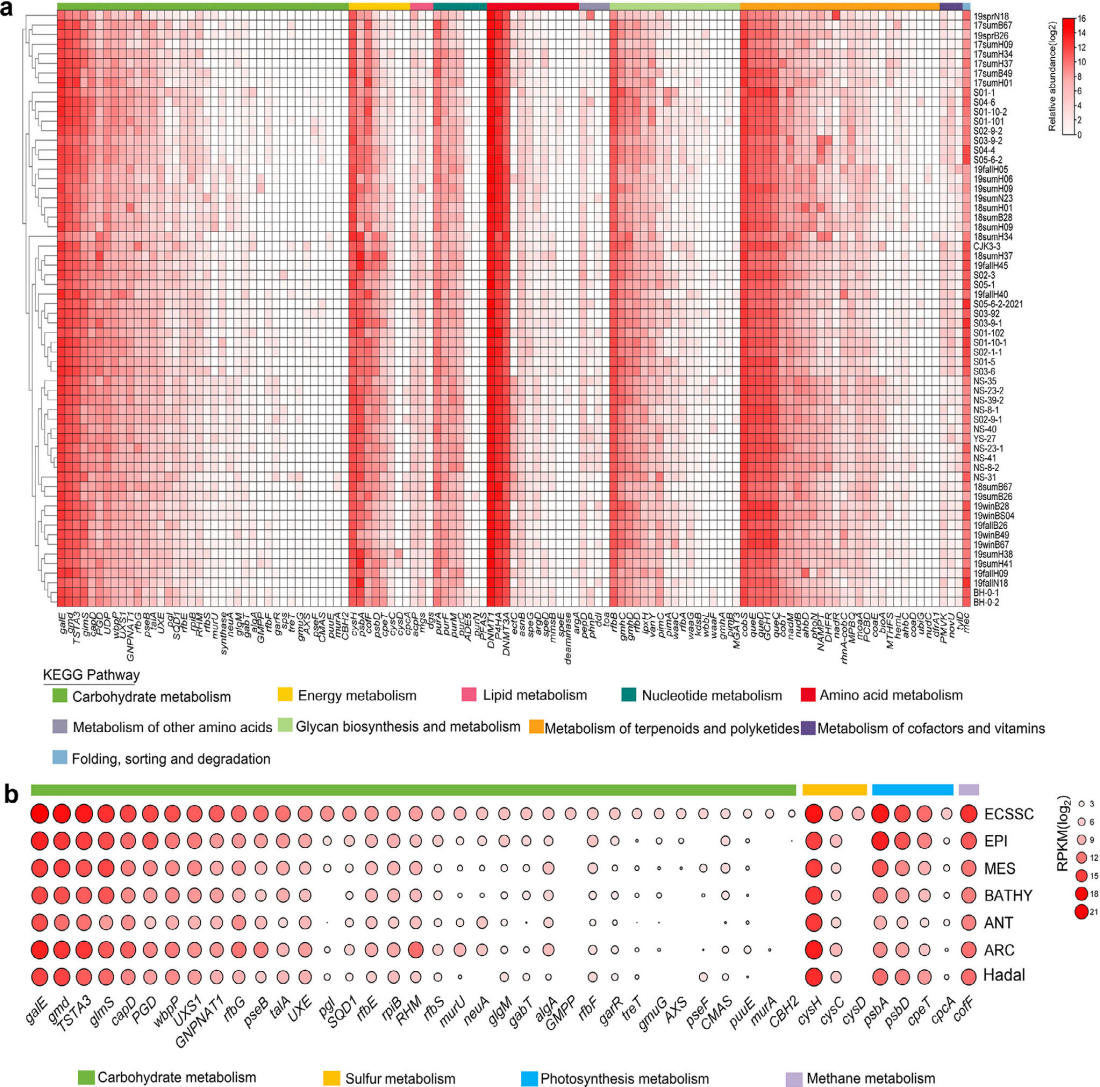
Function and abundance profiles of virus‐encoded auxiliary metabolic genes (AMGs). a) Relative abundance of viral AMGs obtained from viromes dataset in different ECSSC samples. The main metabolic categories are colored according to the legend. b) Relative abundance of carbohydrate, sulfur, photosynthesis and methane metabolism‐related AMGs in the ECSSC, GOV 2.0 (EPI, BATHY, MES, ANT, ARC) and Hadal.

Viruses can affect host metabolism through encoding AMGs.^[^ ^8^ ^]^ To better understand the ecological potentials of viruses in the ECSSC, a comprehensive search of AMGs in viral genomes was performed and their relative abundances were calculated. Based on VIBRANT annotations, a total of 20146 genes were identified as putative AMGs involved in a variety of metabolic pathways, including those related to the metabolism of cofactor vitamins, carbohydrate and amino acid metabolism (Table S2 and Figure S5, Supporting Information). These AMGs likely manipulate host metabolism to enhance viral replication.^[^ ^25^ ^]^ The most abundant AMG, DNA cytosine methyltransferase (DNMT1, *dcm*), is conserved across marine viromes and critical for viral life cycles.^[^ ^26^ ^]^ The abundance of AMGs related to carbohydrate metabolism was relatively abundant in the ECSSC, such as genes coding for UDP‐glucose 4‐epimerase (*galE*) and GDP mannose 4,6‐dehydratase (*gmd*) (**Figure** **4**a). These can affect the form of organic carbon by regulating the synthesis and degradation of polysaccharides in the host cell wall.^[^ ^25^ ^]^ In addition, AMGs involved in energy metabolism were identified, with *cysH* being the most abundant, followed by *psbA* and *cofF* (Figure 4a), which were involved in sulfur, photosynthesis and methane metabolism, respectively. Of these, *cysH* encoded phosphoadenosine phosphosulfate reductase, which catalyzed a critical step in assimilatory sulfate reduction.^[^ ^27^ ^]^ The highly abundant photosynthesis‐associated *psbA* that may evolve under distinct selection pressures, could potentially optimize photosynthetic efficiency in infected hosts.^[^ ^28^ ^]^ The methane metabolism‐linked *cofF* was involved in coenzyme F_420_ synthesis for methanogenesis, implicating viral roles in microbial adaptation to dynamic shelf conditions.^[^ ^29^, ^30^ ^]^

Some carbohydrate metabolism‐related AMGs (*pgl*, *glgM*, and *gabT*) were found that were more abundant in the ECSSC than other areas, especially polar, pelagic and hadal regions (Figure 4b). This indicates that viruses influenced organic carbon dynamics in this nutrient‐rich, eutrophic system‐a key driver of global carbon cycling.^[^ ^31^ ^]^ Photosynthesis‐associated AMGs, including *cpeT* (phycobilin synthesis) and *cpcA* (phycocyanin complex formation), showed higher abundance in the ECSSC relative to other regions, potentially enhancing the light energy capture of photosynthetic organisms.^[^ ^32^ ^]^ The distinct metabolic processes observed in the ECSSC, compared to other regions, underscores their central role in driving biogeochemical cycles within this system. The abundance of *cysC* (adenylylsulfate kinase) and *cysD* (adenylylsulfate kinase) were significantly higher in the ECSSC compared to other regions, particularly in the absence of *cysD* detection in other areas (Figure S6, Supporting Information). As algal blooms frequently occur here, viral *cysD* could regulate sulfur metabolism in hosts, impacting bloom dynamics and ecosystem stability.^[^ ^33^, ^34^ ^]^ This underscores the role of ECSSC viruses in biogeochemical cycling, emphasizing the need for more attentions of AMGs in shaping continental shelf seas dynamics.

### 2.5 ARGs and VFs are Enriched in the ECSSC Viruses

**Figure 5:**
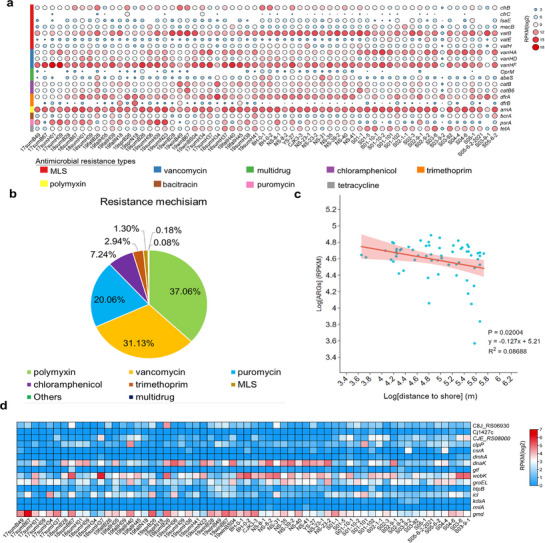
Function and abundance profiles of antibiotic resistance genes (ARGs) and virulence factors (VFs). a) Identification of ARGs encoded in the ECSSC viromes. Heatmap represented the relative abundance of ARGs. The involved different mechanisms of ARGs were shown in the light panel. b) The mean proportion of antimicrobial resistance types in the ECSSC of 62 viromes. c) The relationship distance to shore and normalized abundance of viral ARGs in the ECSSC. The shaded gray region reflects 95% confidence intervals of the fitted regression line. The Pearson's correlation coefficient of the linear regression is presented. Statistical significance of the model was evaluated using a two‐sided F test. d) Identification of VFs encoded in the ECSSC viromes. Heatmap represented the relative abundance of VFs.

As viruses have been recognized as a reservoir of ARGs,^[^ ^35^ ^]^ the composition of ARGs encoded in viruses of the ECSSC was investigated. A total of 220 ARGs were identified, among which *vanHF* (vancomycin resistance), *arnA* (polymyxin resistance) and *vatB* (macrolides‐lincosamids‐streptogramins resistance) were the most abundant ARGs (**Figure** **5**a). Seven resistance mechanisms were identified, with the most abundant being polymyxin (37.06%), vancomycin (31.13%) and puromycin (20.06%) (Figure 5b). It was found that the abundance of ARGs decreased significantly with increasing distance from the shore (Figure 5c). Human activities (57.52%) were showed as the main factors affecting the spatial distribution of ARG abundance. Results presented here indicated that the distribution of viral‐encoded ARG had a distinct “human footprint” characteristic, which suggested they could be used as biomarkers to assess regional anthropogenic pollution.

Based on the functional annotation of the Virulence Factor Database (VFDB), 207 VFs were identified in this study. Among them, the VFs *gmd* and *wcbK* encoding GDP‐mannose dehydrogenase were abundant. These genes may enhance the host immune escape ability of pathogenic bacteria by regulating the capsular polysaccharide biosynthesis pathway (Figure 5d). From the perspective of functional classification, immunomodulatory related virulence factors accounted for the highest proportion, indicating that viruses may significantly affect the interaction between pathogens and host immune system by mediating horizontal transfer of such genes.

### 2.6 Factors Shape Dynamics of Viral Community and Functional Genes in the ECSSC

The dynamics of viral community and functional genes are mainly influenced by microbial community and environmental factors.^[^ ^36^ ^]^ Temperature, salinity, and nutrient levels (NO_3_^−^, NO_2_^−^, NH_4_^+^, PO_4_^3−^ and SiO_3_^2−^) were assessed across 62 stations in the ECSSC (Table S3, Supporting Information). Temperatures ranged from 5.1 to 29.08 °C, while salinity fluctuated from 22.53 to 33.14‰ (g L^−1^). The concentrations of nitrogen (NO_3_^−^, NO_2_^−^ and NH_4_^+^), phosphorus (PO_4_^3−^) and silicate (SiO_3_^2−^) varied from 0 to 22.3, 0.01–0.96, and 1.88–32.86 µmol L^−1^, respectively. Through analysis of environmental factors, it was found that there was significant difference between stations (*P* < 0.05, Figures S7 and S8, Supporting Information). The higher concentration of nitrogen (NO_2_^−^, NO_3_^−^, and NH_4_^+^) in BYS could be attributed to urban wastewater and industrial discharges.^[^ ^37^ ^]^ The average concentration of PO_4_^3−^ was an overall low, which due to the absorption of phosphate by phytoplankton during photosynthesis in surface water.^[^ ^38^ ^]^ The concentration of SiO_3_^2−^ showed a higher level in ECS and lower in BYS, and potentially due to the influx of silicate from the river into the estuary.^[^ ^39^ ^]^

**Figure 6:**
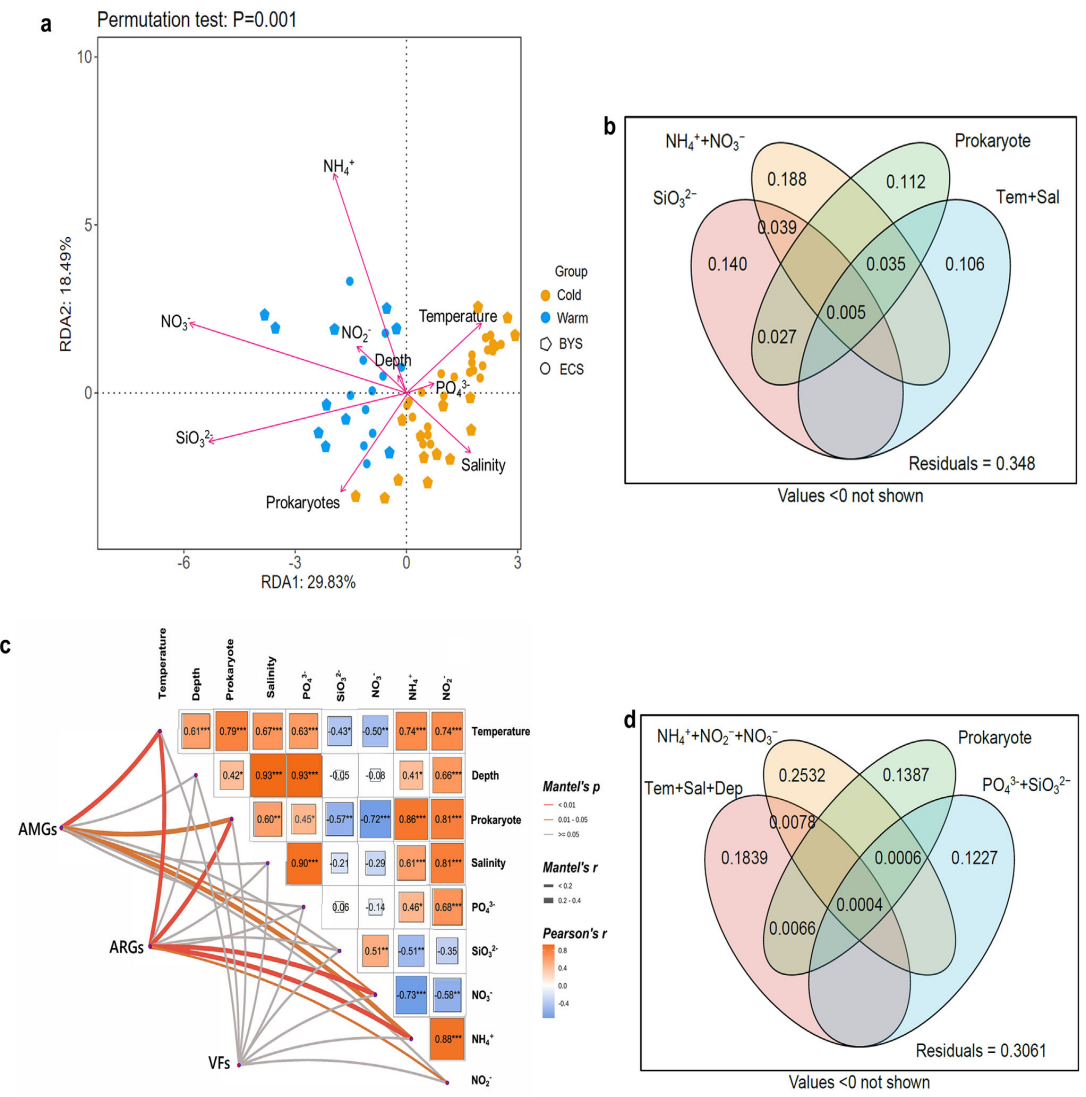
Analysis of the correlation between the viral community structures and functions with the environmental parameters. a) Redundancy analysis (RDA) based on the viral community and environmental parameters. Different colors represent different seasons. Different shapes represent different regions. b) Variation partitioning analysis showing the contributions of environmental factors to the compositional variations of viral communities. c) The Mantel tests showing the relationship between environmental factors with viral community structures and functions. The edge color and width represent the Mantel's r and *p* value, respectively. The color gradient in heatmap represents the Pearson's correlation coefficients between different environmental factors. The stars in the heatmap indicate significance levels: * (*P <* 0.05), ** (*P <* 0.01), and *** (*P <* 0.001). All statistical tests were two‐tailed. d) Variation partitioning analysis showing the contributions of environmental factors to the compositional variations of viral functional dynamics.

Redundancy analysis (RDA) showed that microbial community and environmental factors explained 46.1% of viral community variation (axes 1: 29.8%, axis 2: 18.5%) (**Figure** **6**a). The distribution of viral communities in the BYS was positively related to Prokaryote and SiO_3_^2−^, while those in the ECS were positively related to temperature and negatively to NO_3_^−^ and Prokaryote. The higher concentration of nitrogen sources (NO_2_^−^, NO_3_^−^ and NH_4_^+^) in the BYS could be attributed to urban wastewater and industrial discharges.^[^ ^37^ ^]^ The viromes in warm seasons were positively correlated with NO_3_^−^, NH_4_^+^, Prokaryote and SiO_3_^2−^ while those in cold seasons were positively related to salinity and temperature. Nitrogen and Prokaryote emerged as key drivers of viral family distributions. A correlation analysis (Pearson's correlation coefficient) was performed to explore the relationship between environmental factors and the most abundant 20 viral families (Figure S9a, Supporting Information). NO_2_^−^, Prokaryote and SiO_3_^2−^ exhibited positive correlations with most of the viral populations. Conversely, NO_3_^−^ was significantly negatively correlated with viral abundances, for most of the viral families. Variance partitioning analysis (VPA) of key variables (Prokaryote, NO_3_^−^, SiO_3_^2−^, NH_4_^+^, temperature and salinity) found that 67.6% of viral community variation (*P* < 0.01) could be explained by measured environmental parameters values, with independent effects (51.3%) dominating over interactions (10.6%; Figure 6b). Nitrogen speciation emerged as the primary regulator, with NO_3_^−^ and NH_4_^+^ jointly accounting for 18.8% of independent variance—2.3‐fold higher than other nutrients. Random Forest analysis further ranked NO_3_^−^ as the strongest predictor of viral diversity (IncMSE% = 31.7±2.1%, *P <* 0.05; Figure S9b, Supporting Information), consistent with its role as the primary electron acceptor in microbial respiration.

Mantel test suggested that the functional genes were significantly correlated with environmental factors and microbial community (*P <* 0.01; Figure 6c). AMGs exhibited tightest correlations with temperature (*r* = 0.61) and NH_4_^+^ (*r* = 0.57), mirroring their involvement in nutrient acquisition systems. ARGs showed broader associations (temperature: *r* = 0.59; Prokaryote: *r* = 0.54; NO_3_^−^: *r* = 0.52; NH_4_^+^: *r* = 0.51), possibly reflecting horizontal gene transfer facilitation under nutrient stress. VFs displayed niche specificity, correlating solely with NH_4_^+^ (*r* = 0.31, *P <* 0.05), potentially driven by ammonium‐enhanced host lysis efficiency. VPA resolved regulatory hierarchies: 68.3% of variance was environmentally explained (*P<*0.01), with inorganic nitrogen contributing 27.1% independently (Figure 6d). Random Forest modeling further revealed environmental controls on viral functional dynamics (Figure S9c, Supporting Information). Strikingly, NO_3_^−^ emerged as the predominant predictor of functional gene variation (IncMSE = 31.5±2.1%).

## 3 Discussion

Viruses are thought to be the most widely distributed marine organisms and play important roles in nutrient cycling and genetic exchange in marine ecosystems.^[^ ^4^ ^]^ Yet their diversity and ecological roles in continental shelf seas remain poorly characterized. It has recently been shown that viral communities derived from viromes had higher species richness and total viral genome abundances compared to those derived from metagenomes.^[^ ^40^ ^]^ Therefore, a large‐scale DNA virus community in the ECSSC, collected between 2017 and 2022 was explored based on viromes. We obtained 7659737 viral contigs (≥1.5 kb), representing the largest current dataset of continental shelf seas viromes. We found that over 55% of the viral genes lacked matches in the GOV2.0 database. Additionally, 57.7% of the DNA viruses could not be classified and exhibited low sequence similarity with the viral reference sequences available in the current database. Furthermore, 76.77% of the virus clusters were unique to ECSSC viruses, implying the high novelty of the DNA viruses in the ECSSC. Overall, these findings underscore the significance of ECSSC as an underexplored region, emphasizing the need for further comprehensive studies in this region.

This virome dataset was generated for these largely unexplored ECSSC and this established an important viral dataset for further ecological and evolutionary studies. It is critical ecological ecotones between land and open ocean, one of the most productive marine ecosystems, and plays a key role in global climate change.^[^ ^41^ ^]^ This study revealed significant spatiotemporal variations in the diversity of DNA virus community structures within the ECSSC. Notably, the viral diversity during the warm season was markedly higher than that observed in the cold season (*P* < 0.05), aligning with findings from other marine regions globally.^[^ ^42^ ^]^ The higher water temperatures during summer facilitate phytoplankton proliferation, which subsequently enhances host microorganism activity and creates favorable conditions for viral replication and transmission. Conversely, winter's low temperatures coupled with vigorous water mixing may alter both the structure and function of viral communities. For instance, research on virus distribution in the Yellow Sea during spring indicates that hydrological dynamics can drive spatial heterogeneity among viruses. A significant difference in diversity indices was noted between the BYS and ECS. The greater diversity observed in the Bohai Sea may be attributed to terrestrial nutrient inputs that provide abundant resources for viral hosts.^[^ ^43^ ^]^ In contrast, strong ocean currents such as the Kuroshio Current and Taiwan Warm Current influence the ECS, potentially leading to homogenization of microbial niches among hosts, thereby affecting viral diversity. These findings are consistent with observations made in both the North Atlantic and Mediterranean regions, suggesting a universal pattern regarding spatiotemporal dynamics within viral communities.^[^ ^44^ ^]^ The spatiotemporal variations of DNA viruses at the family level in the ECSSC were also significant. Specifically, *Phycodnaviridae* dominated warm seasons (algal host proliferation),^[^ ^45^ ^]^ while *Iridoviridae* was enriched in the Bohai and Yellow Seas, reflecting aquaculture impacts.^[^ ^46^ ^]^ *Iridoviridae* belongs to NCLDVs and is one of the important viral pathogens of fish and crustaceans. This study highlighted the complexity and diversity of ECSSC viral communities and provided valuable insights for further research on viral their role in marine ecosystems.

Predicting the host of viruses and comprehending the interactions between phages and bacteria are pivotal to the understanding of the ecology and function of microbial communities.^[^ ^5^ ^]^ Here, it was found that viruses in the ECSSC may infect a range of 33 bacterial and 10 archaeal phyla levels, such as Pseudomonadota, Bacteroidota and Bacillota. This is consistent with previous studies on coastal environments.^[^ ^24^, ^47^ ^]^ Interestingly, we identified 2491 vOTUs linked to archaea, such as Nanoarchaeota and Thermoplasmatota. As our understanding of archaeal viruses remains nascent, current knowledge is predominantly anchored to metagenomic assembled genomes (MAGs) linked to uncultivated taxa from extreme marine ecosystems, with over 75% of characterized archaeal viruses deriving from hypersaline environments and deep‐sea hydrothermal vents. Hence, this study provides important genome resources for understanding marine archaeal viruses. The results underscore the need for further research on virus–host interactions in continental shelf environments to better understand their role in microbial diversity and ecosystem function.

Although viruses are usually not directly involved in biogeochemical cycles, they can promote the release of nutrients from hosts by lysis and enhance host metabolism by encoding critical functional genes. A number of studies have demonstrated that viruses frequently carry AMGs to regulate the corresponding biogeochemical cycling processes in different habitats.^[^ ^48^ ^]^ In this study, several AMGs associated with energy metabolism were found in the ECSSC, including those related to photosynthesis and sulfur and methane metabolism. In addition, the relative abundances of AMGs were significantly correlated with corresponding environmental factors, providing compelling evidence for the contribution of viruses to a range of biogeochemical cycles. The comprehensive analysis found that a diverse array of viral AMGs were implicated in crucial metabolic pathways, underscoring the significant influence of viruses on biogeochemical cycles in continental shelf seas. The predominance of DNA cytosine methyltransferase (DNMT1, *dcm*) among identified AMGs aligns with previous studies that underscore its ubiquitous presence across diverse viromes.^[^ ^49^ ^]^ This suggests a conserved function in viral genome regulation, potentially shaping viral lifecycle dynamics in the ECSSC. Viruses appear to significantly impact carbon metabolism, as evidenced by the abundance of carbohydrate metabolism‐related AMGs such as UDP‐glucose 4‐epimerase (*galE*) and GDP mannose 4,6‐dehydratase (*gmd*). These genes likely influence organic carbon form through host cell wall modification, a process more prominent in the ECSSC than in polar or pelagic zones. This provides compelling evidence that viral modulation of host metabolism has broader implications for local and global carbon cycling. The detection of sulfur metabolism‐related AMGs highlights a unique viral contribution to sulfur cycles, potentially influencing algal bloom dynamics prevalent in the nutrient‐rich waters of the ECSSC.^[^ ^50^ ^]^ The particular abundance of *cysD*, absent in other regions, suggests viruses may play a pivotal role in regulating sulfur reduction processes, thus affecting ecosystem sustainability and biological productivity.^[^ ^51^ ^]^ Additionally, the presence of AMGs linked to photosynthesis, such as *psbA*, *psbD*, and genes involved in phycobilin synthesis, demonstrates the influence of viruses on enhancing photosynthetic capacity and efficiency.^[^ ^38^ ^]^ This was especially relevant in the ECSSC surface environments where algal blooms drive rapid biological activity.^[^ ^38^ ^]^ This finding indicate that viruses may introduce novel photosynthetic variants, optimizing energy conversion in virus‐infected cells and thereby influencing primary productivity. The presence of genes related to methane metabolism, including *cofF*, emphasize a potential viral role in greenhouse gas dynamics.^[^ ^52^, ^53^ ^]^ Given these findings, it is clear that viral AMGs play non‐trivial roles in modulating key biogeochemical processes within the ECSSC. Future research should focus on genome context assessments and functional analyses to elucidate the mechanistic underpinnings of AMG‐mediated host‐virus interactions. These insights will be crucial for understanding how viruses shape ecological and climate‐related processes in marine ecosystems.

In recent years, with the increasing overuse of antibiotics, the problem of drug resistance and virulence evolution of bacterial pathogens has become increasingly prominent and the focus of global concern.^[^ ^54^ ^]^ In this context, study of the role of virus‐mediated horizontal gene transfer in the transmission of bacterial ARGs and VFs is of great significance for revealing the evolution mechanism of pathogens and formulating prevention and control strategies.^[^ ^45^, ^55^, ^56^ ^]^ In the ECSSC, an area with intensive human activity, frequent terrestrial inputs, aquaculture activity, and climate change may increase the complexity of virus‐host interactions, thus providing a potential environmental hotspot for the transmission of VFs.^[^ ^57^ ^]^ In this study, a range of ARGs were found in the ECSSC, among which *vanHF* genes (vancomycin‐resistant) were the most abundant. In addition, ARGs associated with polymyxin, vancomycin and puromycin were also abundant. These results suggest that viruses may contribute to the spread of ARGs through horizontal gene transfer, thereby exacerbating the problem of antibiotic resistance.^[^ ^58^, ^59^, ^60^ ^]^ Viruses play an important role in the virulence evolution of pathogens.^[^ ^61^ ^]^ In this study, 207 VFs were identified by comparison with the VFDB. Of these, *gmd* was the most abundant, and its function was involved in the synthesis of bacterial capsular polysaccharides, thereby enhancing the immune escape ability of pathogens. In addition, *wcbK*, which is involved in environmental adaptation, was also highly abundant. This suggests that viruses may affect interactions between pathogens and the host immune system by mediating the horizontal transfer of VFs.^[^ ^60^ ^]^ These findings highlight that viruses‐encoded ARGs and VFs functions are an associated potential health risk and deserve much more attention. As DNase treatment was not performed in this study,^[^ ^50^, ^62^ ^]^ it may cause some non‐viral DNA contamination. This is a particular concern for the ARGs and VFs, as their presence might potentially be attributed to contaminating bacterial DNA rather than bona fide viral genomes. However, we used VirSorter2, DeepVirFinder, and VIBRANT to identify viral sequences on the basis of genomic signatures, which effectively removed most bacterial genome fragments and greatly reduced the impact of contamination. This made the discussed genetic content primarily representative of real viral sequences. In addition, microbial metagenomes were also be used to identify viral contigs during past decade.^[^ ^22^ ^]^ This problem should be paid special attention for the viral identification from microbial metagenomes. In future, the virome studies employing DNase treatment, will help to further validate the viral identification.

The flow of total nitrogen into rivers from human waste and agricultural runoff could potentially double.^[^ ^3^ ^]^ Agriculture comprises approximately two‐thirds of the Bohai Bay basin land use, leading to a potential future increase in the use of fertilizers.^[^ ^63^ ^]^ In this study, salinity was higher in BYS and lower in ECS. This may have been due to an influx of freshwater into ECS, while BYS was more influenced by ocean currents and tides.^[^ ^64^ ^]^ The higher concentration of nitrogen (NO_2_^−^, NO_3_^−^ and NH_4_^+^) in BYS could be attributed to urban wastewater and industrial discharges.^[^ ^37^ ^]^ The average concentration of PO_4_^3−^ was low, which is probably due to the take up of phosphate by phytoplankton during photosynthesis in surface water.^[^ ^38^ ^]^ The concentration of SiO_3_^2−^ showed a higher level in ECS and lower in BYS, potentially due to the influx of silicate from rivers into the estuary.^[^ ^39^ ^]^ These findings suggest that the spatial distribution of nutrients in the ECSSC was directly influenced by anthropogenic activity. Our findings are consistent with previous research that suggests that environmental conditions significantly influence virus abundance in the upper ocean.^[^ ^11^ ^]^ Similarly, our results establish environmental parameters as key predictors of viral community structure, with implications for modeling ecosystem responses to coastal pollution. These findings provide valuable insights into the complex interplay between viruses and environments, which could have significant implications for understanding the ecology and evolution of viral communities in marine ecosystems.

This study presents the first comprehensive analysis of marine viral diversity and ecological potentials in the ECSSC, integrating 310628 vOTUs and 3662956 viral genes from 62 seawater viromes. Comparative analysis with global viromes highlighted the uniqueness of ECSSC viruses, with 76.77% representing novel lineages. The findings suggest close interconnected relationships between viral and microbial communities. Viruses encoded diverse AMGs linked to carbohydrate, sulfur, photosynthesis and methane metabolism, potentially enhancing host metabolic versatility in this eutrophic continental shelf seas system. Notably, dissimilatory sulfate reduction (*cysD*) and photosynthesis‐associated genes (*cpeT*, *cpcA*) were uniquely enriched, implicating viruses in regulating algal bloom dynamics and carbon fixation. In the ECSSC, *vanHF*, *arnA*, and *vatB* emerged as the most abundant ARGs among the 220 ARGs, with their abundance exhibiting a sharp offshore decline. In this study, 207 VFs were found in the ECSSC, among which immunomodulatory VFs accounted for the highest proportion, suggesting that virus‐mediated horizontal gene transfer may significantly affect the pathogen‐host immune interaction. Environmental factors, notably nitrogen and silicate, were significantly correlated with viral community composition and functional genes. This study provides a blueprint for understanding how marine viruses in continental shelf seas mediated biogeochemical cycles and ecosystem resilience, with implications for predicting marine responses to environmental changes.

## 4 Experimental Section

### Sample Collection and Environmental Parameters

The 62 virome seawater samples were collected from the eastern continental shelf seas of China, including Bohai and Yellow Sea (BYS) and East China Sea (ECS) during 2017‐2022. Seawater samples (20 L) were collected using Niskin bottles attached to a rosette frame, which also recorded the temperature, salinity and depth with the SBE‐9 plus CTD sensor (SBE 911; Seabird Electronics). Sub‐samples of 100 mL were taken from each site for nutrient analysis (NO_3_^−^, SiO_3_^2−^, PO_4_^3−^, NO_2_^−^ and NH_4_^+^) using an on‐board nutrient autoanalyzer (SKALAR SAN plus, the Netherlands). The seawater samples were filtered through a 140 mm diameter cellulose membrane with a pore size of 3 and 0.22 µm to remove non‐viral particles such as micro‐plankton and bacteria.^[^ ^65^ ^]^

### Viral Concentration, DNA Extraction, and Sequencing

Virus enrichment was performed by adding 2 mL of 10 g L^−1^ FeCl_3_ solution to seawater filtered with a 0.2 µm filter.^[^ ^66^ ^]^ After 50 min incubation at 25 °C, samples were passed through polycarbonate membrane filters (0.8 µm; Millipore) and stored at 4 °C until further processing.

The concentrated membranes of filtered virus particles were resuspended in 0.1 m EDTA, 0.2 M MgCl_2_ buffer (pH 6.0) at 4 °C. The viruses were concentrated to about 400 µL by centrifugal ultrafiltration. DNA was extracted by QIAamp DNA mini kit (Qiagen) following the provided instructions and stored at ‐80 °C. High‐throughput sequencing of each viral DNA sample was performed by Novogene (Beijing, China) using Illumina NovaSeq 6000 (pair end sequencing, 2 × 150 bp).

### Viral Contigs Assembly and Identification

The raw reads were removed from the adapters using Cutadapt,^[^ ^67^ ^]^ and high‐quality reads were assembled using MEGAHIT v1.2.9.^[^ ^68^ ^]^ Contigs ≥1.5 kb from virome assemblies were used to recover viral sequences. Three mainstream pipelines, including Virsorter2 v2.2.3,^[^ ^69^ ^]^ DeepVirFinder v1.0,^[^ ^70^ ^]^ and VIBRANT v1.2.1,^[^ ^71^ ^]^ were used to identify viruses from co‐assembled contigs. Then, a more precise procedure was used to screen and retain viral genomes according to the following criteria: i) high confidence level (score ≥0.7 and had hallmark genes) of VirSorter2 (parameters: –keep‐original‐sequence), ii) identified by VirSorter2 (score ≥0.5), DeepVirFinder (score ≥0.7 and *P* ≤0.05), and VIBRANT simultaneously. iii) identified by any two of VirSorter2 (score ≥0.5), DeepVirFinder (score ≥0.7 and *P* ≤0.05), and VIBRANT, and further screened by CheckV v1.0.1^[^ ^72^ ^]^ with at least one viral hallmark gene and the ratio of the cell host gene to the viral gene was less than one. The provirus regions were extracted from recovered viral genomes based on the CheckV contamination estimates.

The detected viral contigs were classified into vOTUs if they shared 95% nucleotide identity across ≥80% of shorter viral contigs using CD‐HIT v4.8.1 (parameters: ‐c 0.95 ‐G 0 ‐M 0 ‐aS 0.8 ‐T 8 ‐n 9).^[^ ^73^ ^]^ The open reading frames (ORFs) of vOTUs were predicted by prodigal with default parameters, and searched against the NR protein database using DIAMOND.^[^ ^74^ ^]^ The Prodigal 2.6.3 (‐p meta) software was used to predict viral genes in viral genomes after removing host contamination. The representative genome of each vOTU (≥1.5 kb) was used as input to CheckV to evaluate genome completeness. The vOTUs (≥5 kb) and circular genomes (1.5–5 kb) were used for subsequent analyses. The lifestyle of the high‐quality viruses was predicted by VIBRANT using the default parameters.^[^ ^71^ ^]^

### Taxonomic Classification, Calculating Relative Abundances and Diversity

The viral taxonomy identification of the vOTUs was annotated using geNomad v1.5.1^[^ ^75^ ^]^ and VITAP^[^ ^76^ ^]^ based on sequence similarity fitting and classification extension utilizing a bipartite graph. The vOTUs that could not be assigned into viral taxonomy were manually searched against the NCBI database.

The clean reads from each sample were mapped to vOTUs using bowtie2^[^ ^77^ ^]^ and counted using CoverM v0.3.1 (https://github.com/wwood/CoverM) (parameters: ‐min‐read‐percent‐identity 0.95 ‐min‐read‐aligned‐percent 0.75). Subsequently, the relative abundances of the vOTUs were determined as RPKM (reads per kilobase per million mapped reads) values. Alpha diversity with Shannon index was calculated using the R package vegan. Non‐metric multidimensional scaling (NMDS) analysis was used to reveal viral β‐diversity from vOTUs based on Bray‐Curtis distance by the function vegdist (method “bray”).

### Comparison of Viral with Other Environmental Virome and Network Analysis

To investigate the uniqueness of the viral community in the ECSSC, this study employed the standardized clustering pipeline from the CheckV database^[^ ^72^ ^]^ to perform comparative analysis. Our viral sequences were clustered against two reference datasets: 1) viral genomes (≥10 kb) from the GOV 2.0 database, and 2) NCBI viral sequences, using thresholds of 95% average nucleotide identity (ANI) and 85% aligned fraction for cluster delineation. Quantitative assessment of cluster sharing with reference sequences was subsequently conducted. Furthermore, a comprehensive database incorporating 3842418 complete and high‐quality viral reference sequences from aquatic habitats in the IMG/VR v4 database was established.^[^ ^21^ ^]^ Whole‐genome nucleotide similarity comparisons between vOTUs and this composite database were performed using BLASTn (e‐value ≤ 1×10^−5^). Read mapping against GOV2 reads including 154 samples using bowtie2 and counted using CoverM v0.3.1 was further performed.

Viral ORFs predicted from the ECSSC viruses were compared against the whole GOV 2.0 protein database using blastp with the following cutoffs: e‐value better than 1×10^−5^ and query coverage and identity values ≥50%.

Viral network interaction showing taxonomic assignment and relatedness of viruses from the ECSSC with other viruses was performed with vConTACT2.^[^ ^78^ ^]^ For this analysis, the high‐quality viral genomes (12518 vOTUs) was compared, including coastal, terrestrial, oceanic, and wastewater environments in the IMG/VR v4.0 databases. Viral proteins were compared through all‐verses‐all blastp with an E‐value threshold of 10^−5^ and 50 for bit score.^[^ ^78^ ^]^ A similarity score for each pair was calculated as the negative logarithmic score by multiplying the hypergeometric similarity *P* value by the total number of pairwise comparison using vConTACT2 (https://bitbucket.org/MAVERICLab/vcontact2). Cytoscape was used to visualize the gene sharing network,^[^ ^79^ ^]^ and Venn diagram was used to show the number of common and unique VC in each habitat.

### Identification of AMGs, ARGs and VFs, and Host Prediction

The AMGs identified by VIBRANT in the above section were first filtered according to the following criteria^[^ ^71^ ^]^: 1) Edge‐located AMGs (AMGs located at either end of a scaffold) were filtered. 2) AMGs that had any KEGG v‐score or Pfam v‐score (assigned by VIBRANT) ≥1 was filtered. 3) AMGs with flanking genes (four genes on either the upstream or downstream sites) having a KEGG v‐score <0.25 were filtered.^[^ ^80^ ^]^ To generate the abundance profiles for AMGs, clean reads were mapped to the virome using bowtie2, and the RPKM values of each gene were calculated. The sum of the RPKM values of genes with the same KO annotations was used to represent the relative abundance of each gene category.

The ARGs and VFs were identified by predicted ORFs against the database of Structured Antibiotic Resistance Genes (SARG)^[^ ^81^ ^]^ and Virulence Factor Database (VFDB)^[^ ^82^ ^]^ (‐query‐cover 70, ‐id 80, ‐e‐value 1 × 10^−5^). To calculate the relative abundance of ARGs, clean reads were mapped to the viromes using coverM and RPKM values were calculated for each gene. The heatmap for antimicrobial resistance genotypes was generated using TBtools.^[^ ^83^ ^]^

iPHoP v1.2.0 (Integrated Phage Host Prediction), a novel method for predicting potential hosts of vOTUs, utilizes a combination of Integrated Microbial Genomes (IMG), Genomes from Earth's Microbiomes (GEM), and Genome Taxonomy Database (GTDB) to facilitate accurate predictions.^[^ ^84^ ^]^ This approach is used to predict the host range of viruses by integrating genomic sequence, protein sequence, and host genomic information. All virus‐host pairs for which the confidence score is higher than the selected cutoff (default = 90) is included, so each virus may be associated with multiple predictions. After the highest score was manually selected, the final hosts of viruses were obtained.

### Comparison to Specific AMGs from Other Habitats

To investigate the distribution and abundance of AMGs associated with carbohydrate, sulfur, photosynthesis, and methane metabolism across diverse habitats, a comparative analysis was conducted between ECSSC and other environmental datasets, including GOV 2.0 (EPI, BATHY, MES, ANT, ARC)^[^ ^10^ ^]^ and Hadal zone^[^ ^85^ ^]^ viromes. Contigs encoding functional annotations related to carbohydrate, sulfur, photosynthesis, and methane metabolism were identified using the KEGG database. These contigs were subsequently analyzed using CoverM v0.6.1 to quantify their coverage profiles across metagenomic datasets (parameters: ‐p bwa‐mem ‐min‐read‐percent‐identity 0.95 ‐min‐read‐aligned‐percent 0.75 ‐m tpm ‐t 20).

### Statistical Analysis

Redundancy analysis (RDA) was performed in R with vegan using the relative abundance of each vOTU and environmental factors. Then, variation partitioning analysis was performed using the forward selection procedure using the “ordistep” function of the canonical correlation analysis model for vegan packages.^[^ ^86^ ^]^ Random Forest Model in machine learning was used to evaluate the influence of key environmental factors on the changes of viral community structure and functional dynamics.^[^ ^87^ ^]^ Pearson correlation analysis was performed using the corrplot package to reveal the potential relationship between abiotic factors and viral communities.

Heatmaps were generated by the pheatmap R package and TBtools. The significance between samples was calculated using nonparametric Mann‐Whitney U test. LEfSe was conducted in the Galaxy online tool (*P* < 0.01 and LDA score>3). The geographic distances to shore between different sites were calculated using the “geoXY” function of SoDA package.^[^ ^88^ ^]^ The Mantel tests between viral communities and functions and environmental factors were performed with the method of Pearson correlation. All other plots were generated by the ggplot2 package.

## Conflict of Interest

The authors declare no conflict of interest.

## Author Contributions

X.G. and C.G. performed research; X.G. wrote the original manuscript. X.G., H.Y., M.W.W., H.S., and Y.Y. analyzed methodology and formal. D.P.‐E. reviewed the manuscript. Y.L., A.M., and M.W. designed and supervised this project, reviewed the manuscript, and provided funding.

## Supporting information



Supporting Information

Supplemental Table 1

Supplemental Table 2

Supplemental Table 3

## Data Availability

The raw datasets used for analysis in this study are publicly available in the Genome Sequence Archive in National Genomics Data Center, China National Center for Bioinformation / Beijing Institute of Genomics, Chinese Academy of Sciences (GSA: CRA026797). The fasta files containing assembled contigs, identified viral contigs (1.5 kb, 5 kb, and 10 kb separately) and reported AMGs have been deposited to https://figshare.com/articles/dataset/Viral_assembled_contigs_of_continental_shelf_seas/29928503; https://figshare.com/articles/dataset/1_5kb_viral_contigs_of_continental_shelf_seas/29928626; https://figshare.com/articles/dataset/5kb_viral_contigs_of_continental_shelf_seas/29937656; https://figshare.com/articles/dataset/10kb_viral_contigs_of_continental_shelf_seas/29937680; https://figshare.com/articles/dataset/AMGs_Sequences/29940929, respectively.
