# Supplementary material for: Diversity and Ecological Potentials of Marine Viruses Inhabiting Continental Shelf Seas
Source: Adv Sci (Weinh). 2025 Nov 14;13(1):e11707. doi: 10.1002/advs.202511707 (PMC12766990; doi:10.1002/advs.202511707)

**Supplemental materials**

**Fig. S1** **General features of viruses in the ECSSC**. **a** The size and number of viruses in the ECSSC. **b** Classification of viruses according to International Committee of Viral Taxonomy (ICTV database). **c** Proportion of taxonomic phylum identified in the ECSSC viral populations. **d** Classification of lytic and lysogenic viruses in the ECSSC.

**Fig. S2** Accumulation curves of vOTUs. The mean ± SEM values are plotted. Boxes represent the average number of vOTUs, and the error bars represent the SEM.

**Fig. S3** The relative proportions of bacterial and archaeal taxa at phylum level. Only the top ten phyla were presented, while the remaining phyla were categorized as others.

**Fig. S4** Relative abundance of vOTUs in the ECSSC against GOV 2.0 reads.

**Fig. S5** Classification of AMGs into KEGG metabolic categories.

**Fig. S6** The presence or absence of carbohydrate, sulfur, photosynthesis and methane metabolism-related AMGs in the ECSSC, GOV 2.0 (EPI, BATHY, MES, ANT, ARC) and Hadal. Genes that were not detected in a given sample remained blue.

**Fig. S7 Temporal distribution of nutrients in the ECSSC**. **a**) Temperature; **b**) Depth; **c**) Salinity; **d**) NO_3_^-^; **e**) NH_4_^+^; **f**) NO_2_^-^; **g**) SiO_3_^2-^; **h**) PO_4_^3-^.

**Fig. S8 Spatial distribution of nutrients in the ECSSC**. **a**) Temperature; **b**) Depth; **c**) Salinity; **d**) NO_3_^-^; **e**) NH_4_^+^; **f**) NO_2_^-^; **g**) SiO_3_^2-^; **h**) PO_4_^3-^.

**Fig. S9 Analysis of the correlation between the viral community structures and functions with the environmental parameters**. **a** Correlation of the 20 most abundant viral populations at the family level with the environmental factors. Red and blue represent positive and negative correlations between genes and environmental factors, respectively. Significant: * (*P<*0.05), ** (*P<*0.01), and *** (*P<*0.001). Random Forest method was used to assess the importance of environmental factors to the viral communities (**b**) and functional genes (**c**).

**Table. S1** Sample and assembly information of 62 viromes in the ECSSC.

**Table. S2** AMGs informations including gene IDs, annotations, and vOTU association.

**Table. S3** The environmental factors information of the ECSSC.


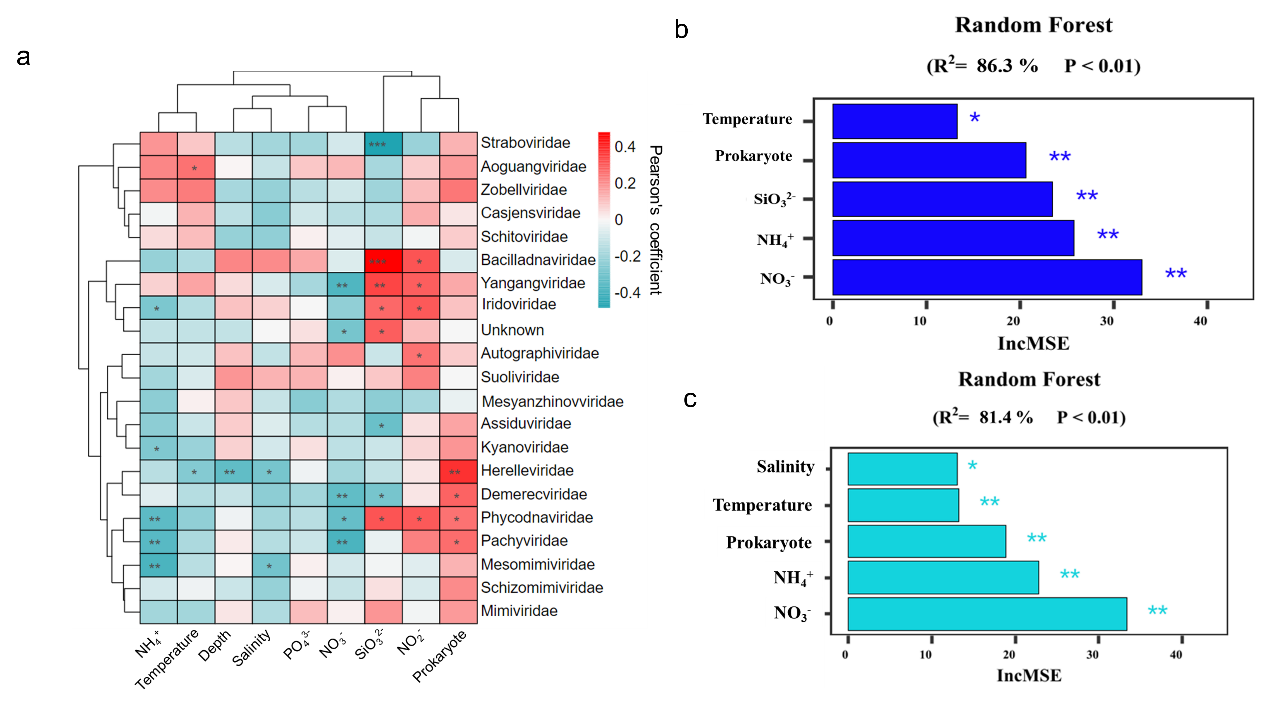

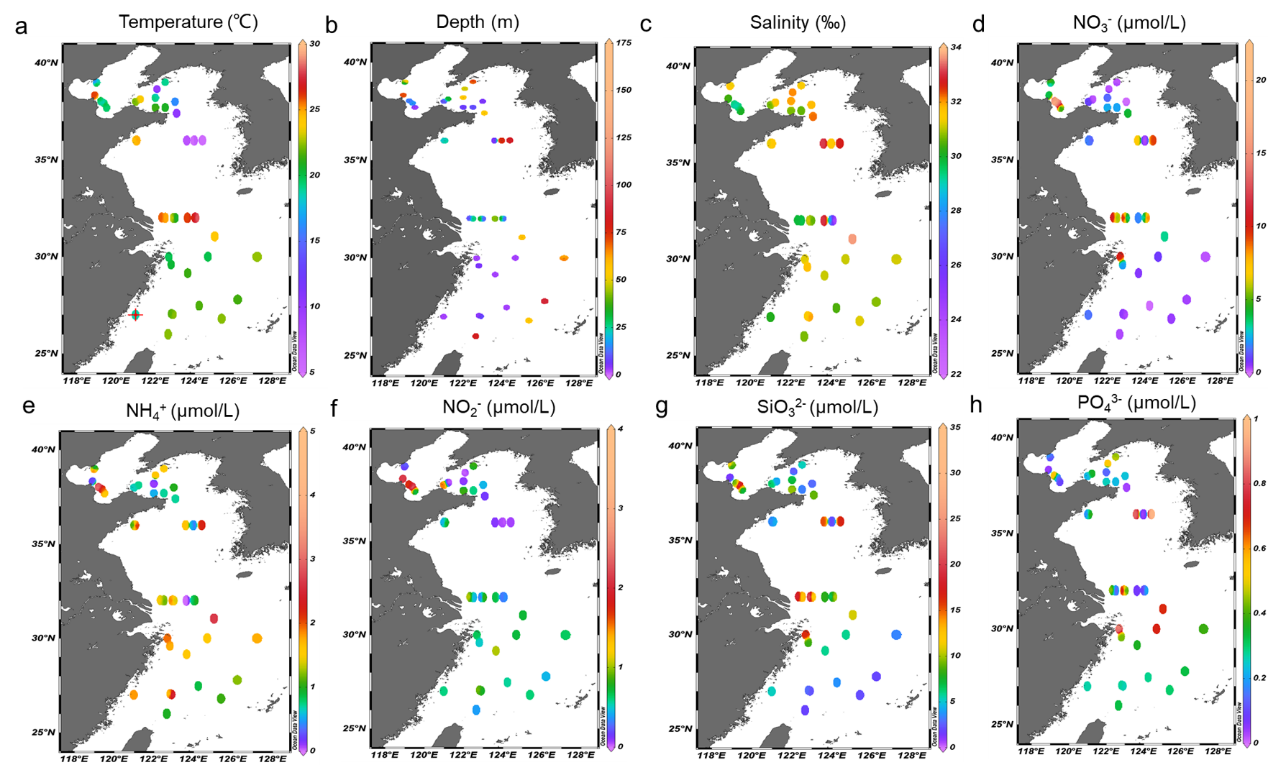

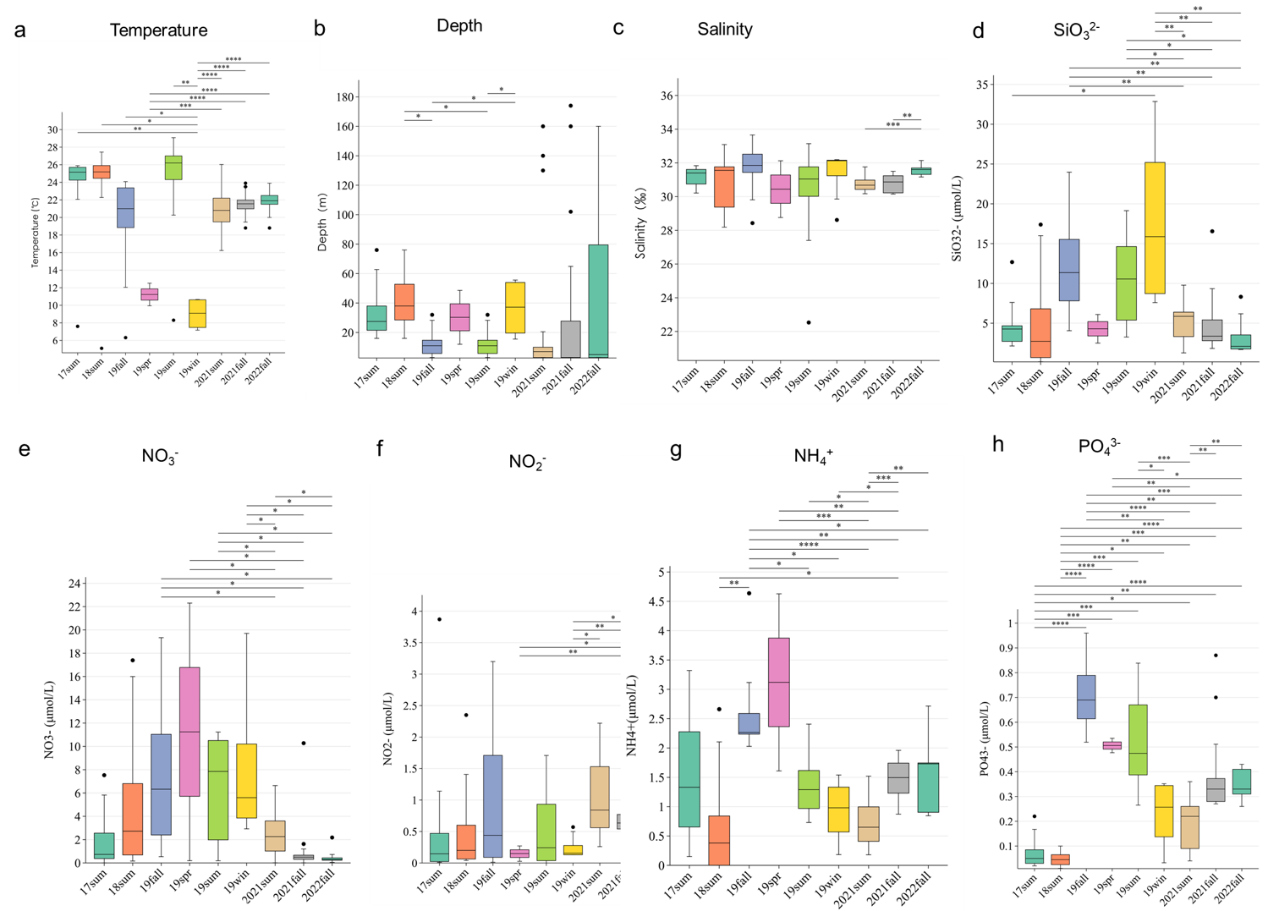

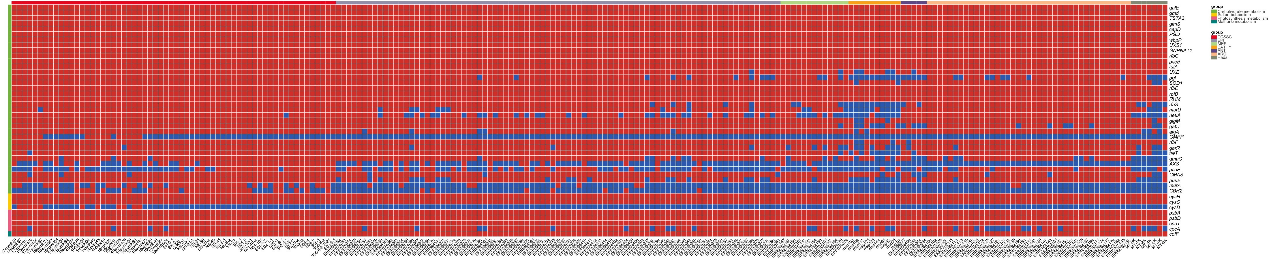

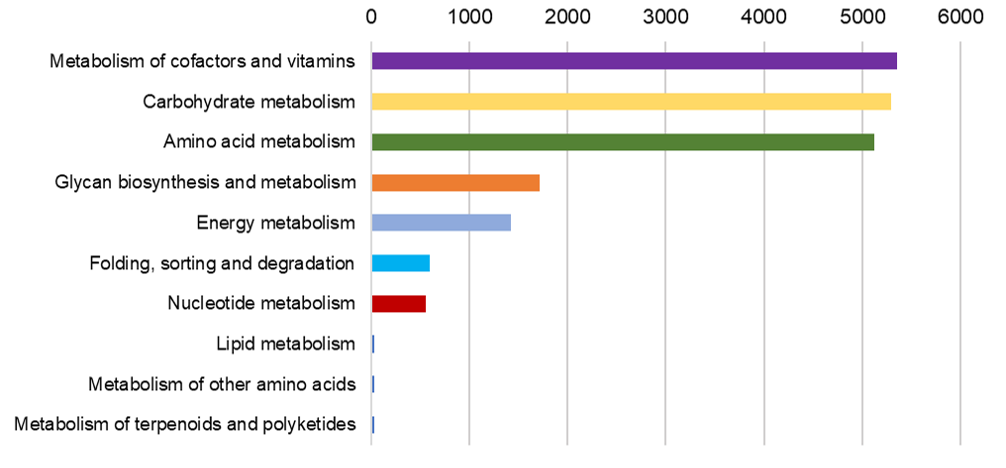

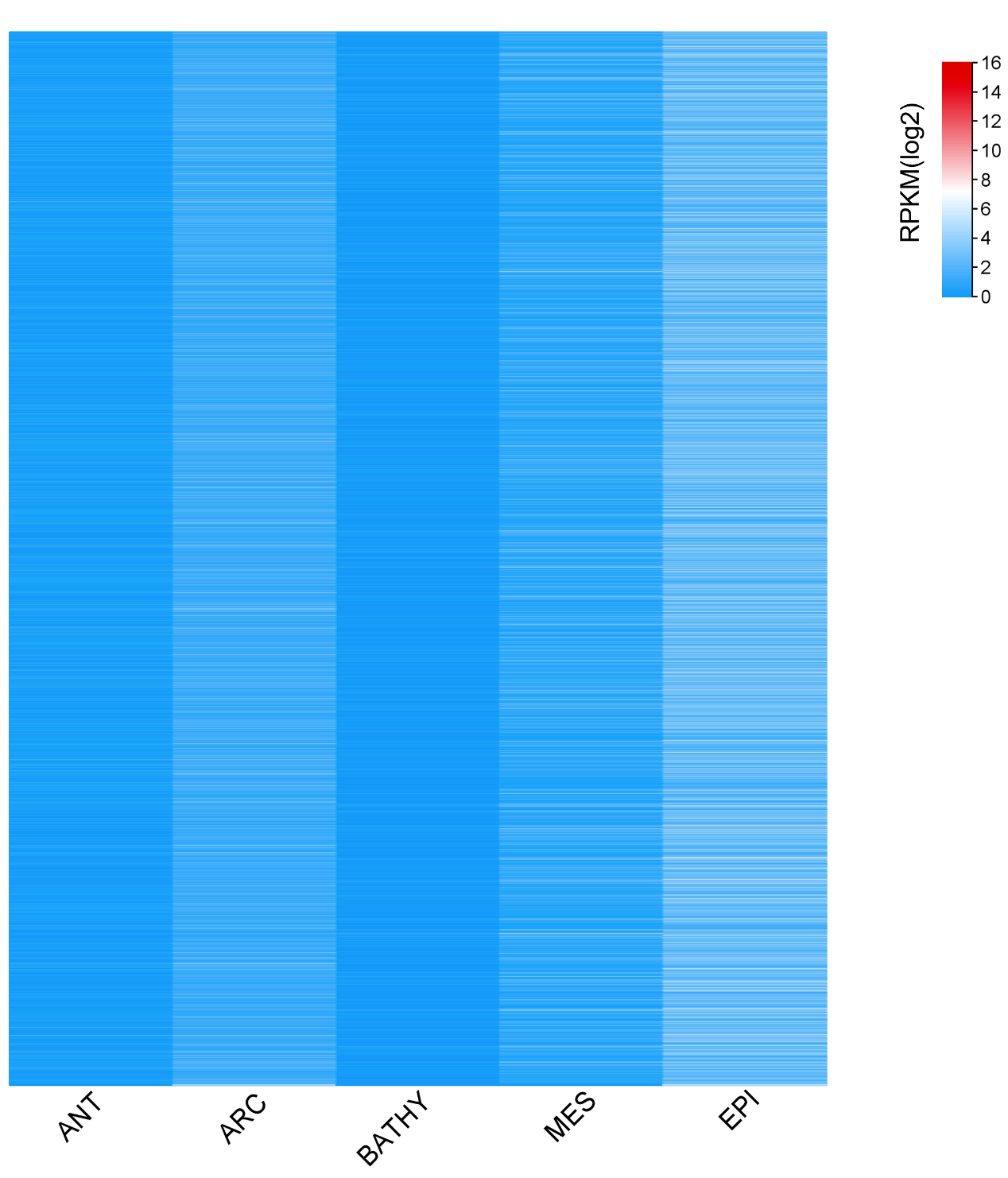

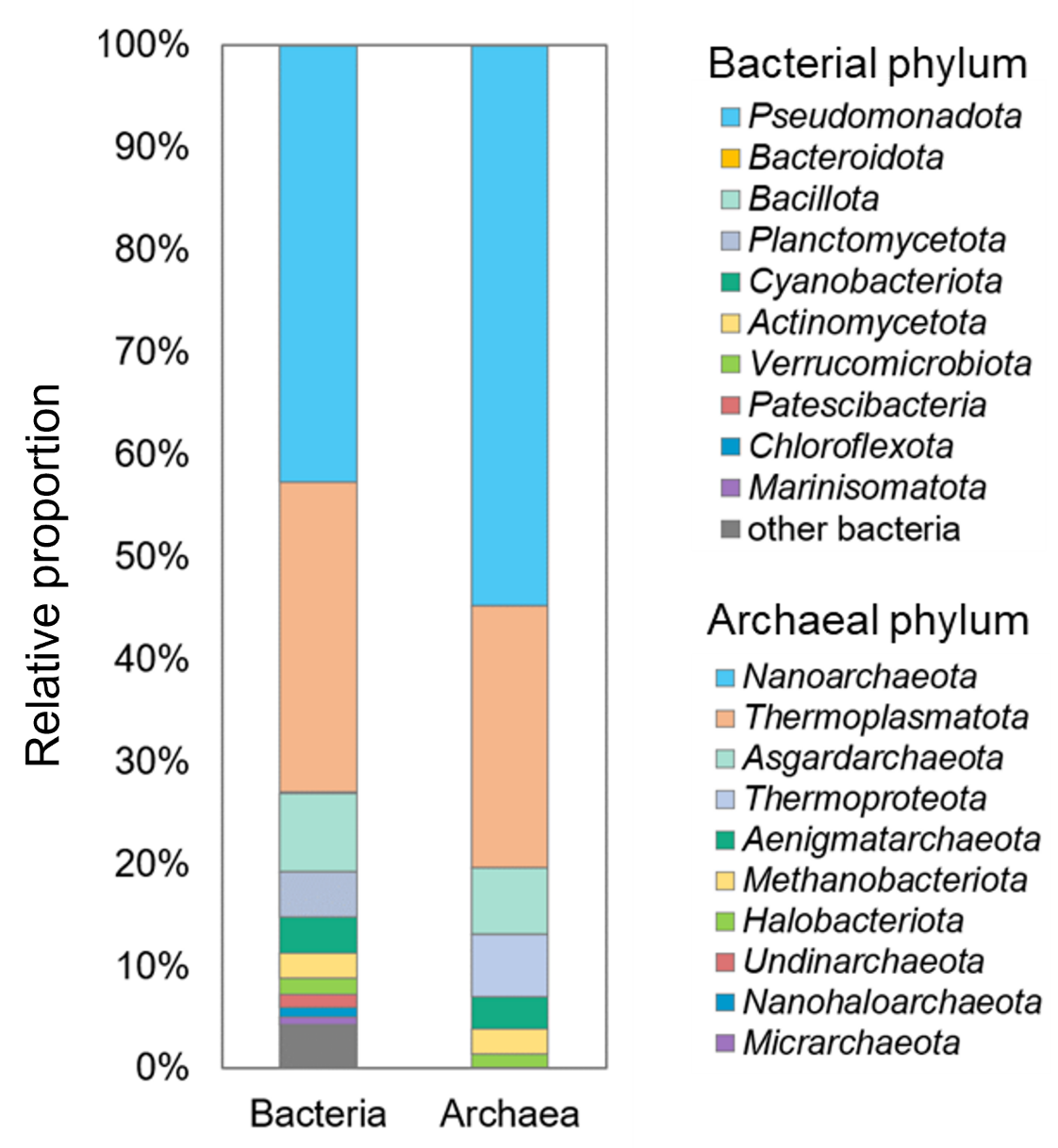

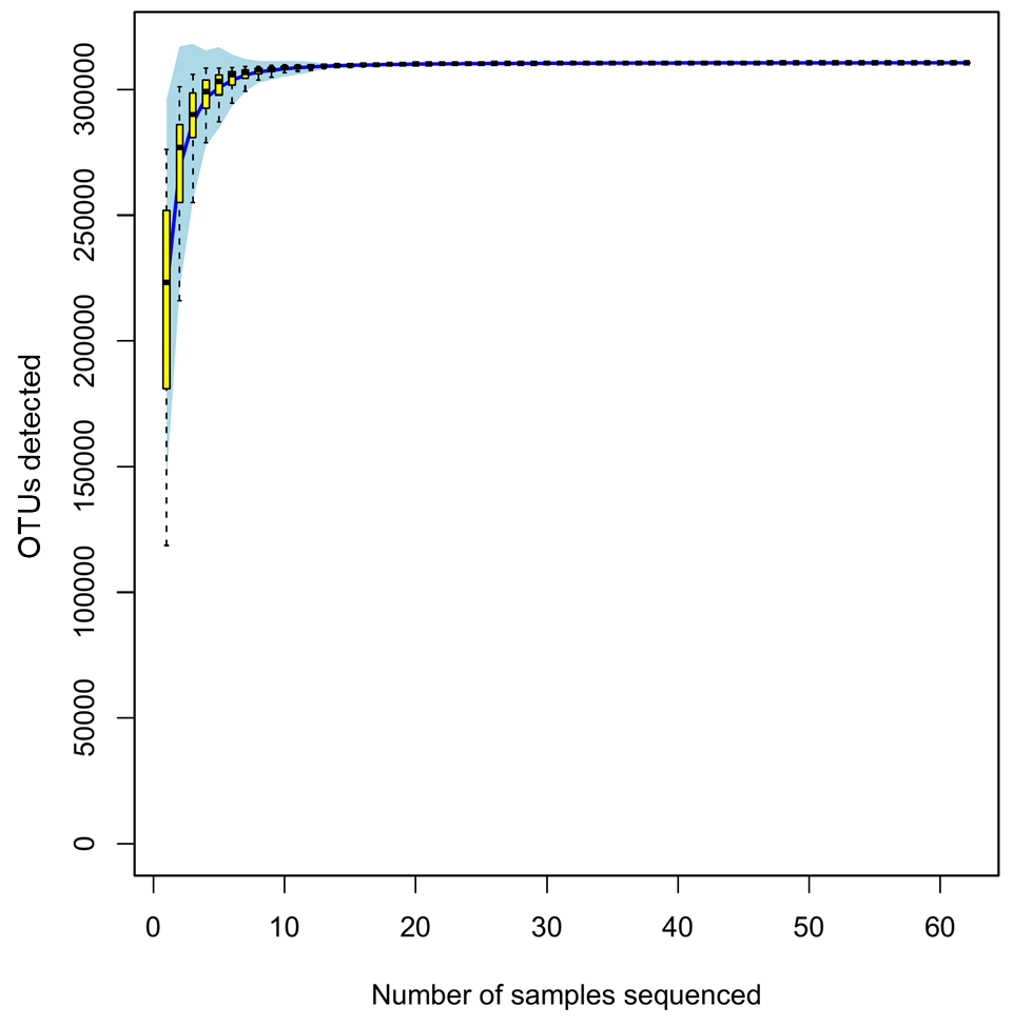

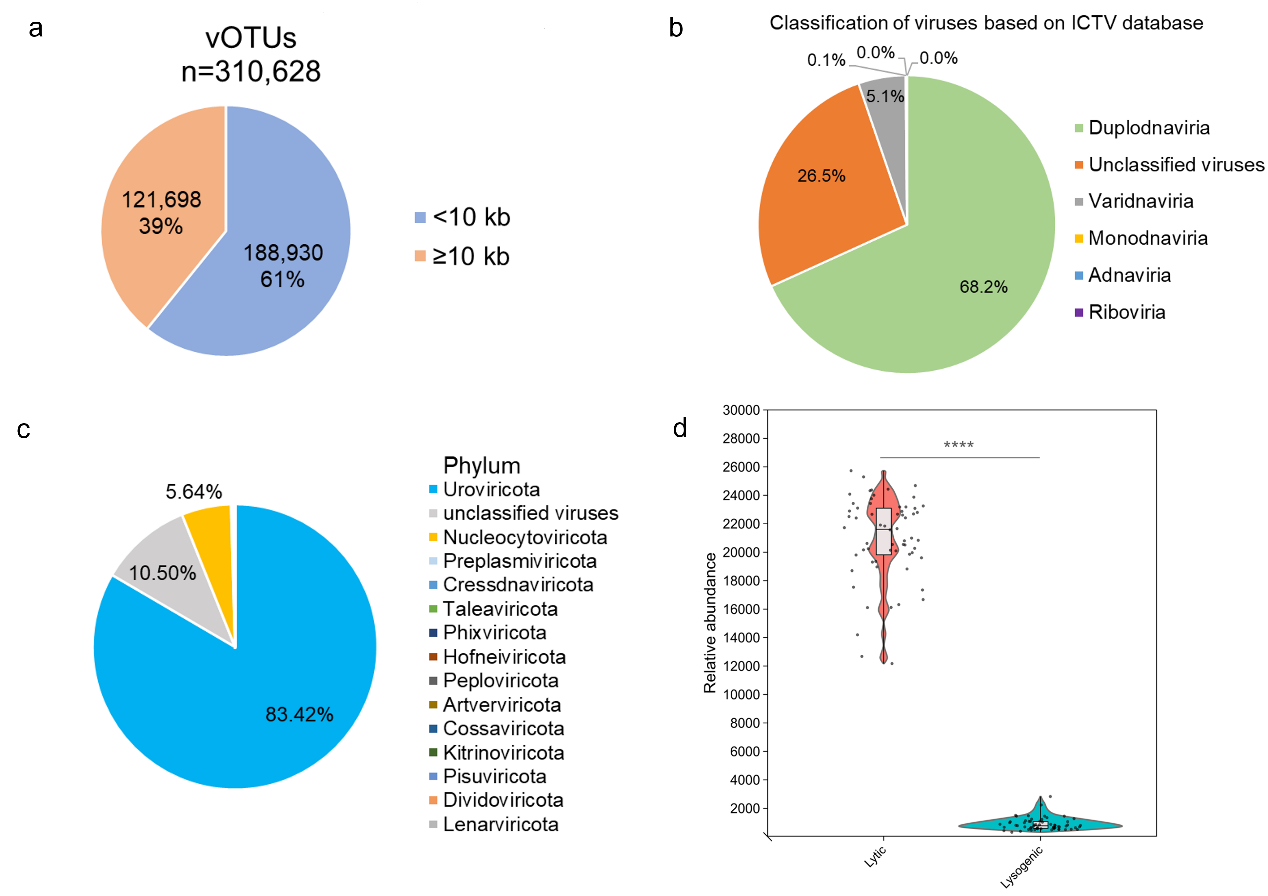

Supplement: Supplementary file 1 — Supporting Information [file ADVS-13-e11707-s003.docx]
